# The Associations Among Individual Factors, eHealth Literacy, and Health-Promoting Lifestyles Among College Students

**DOI:** 10.2196/jmir.5964

**Published:** 2017-01-10

**Authors:** Shu-Ching Yang, Yi-Fang Luo, Chia-Hsun Chiang

**Affiliations:** ^1^ Institute of Education National Sun Yat-Sen University Kaohsiung Taiwan

**Keywords:** individual factors, health-promoting lifestyle, eHealth literacy

## Abstract

**Background:**

eHealth literacy is gaining importance for maintaining and promoting health. Studies have found that individuals with high eHealth literacy are more likely to adopt healthy eating, exercise, and sleep behaviors. In addition, previous studies have shown that various individual factors (eg, frequency of seeking information on health issues, degree of health concern, frequency of eating organic food, and students’ college major) are associated with eHealth literacy and health-promoting lifestyles. Nevertheless, few studies have explored the associations among individual factors, eHealth literacy, and health-promoting lifestyles among college students. Moreover, there is a lack of studies that focus on eHealth literacy as a predictor of psychological health behaviors.

**Objective:**

To examine the associations among various individual factors, eHealth literacy, and health-promoting lifestyles.

**Methods:**

The eHealth Literacy Scale is a 12-item instrument designed to measure college students’ functional, interactive, and critical eHealth literacy. The Health-promoting Lifestyle Scale is a 23-item instrument developed to measure college students’ self-actualization, health responsibility, interpersonal support, exercise, nutrition, and stress management. A nationally representative sample of 556 valid college students in Taiwan was surveyed. A questionnaire was administered to gather the respondents’ background information, including the frequency of seeking information on health issues, the frequency of eating organic food, the degree of health concern, and the students’ major. We then conducted a multiple regression analysis to examine the associations among individual factors, eHealth literacy, and health-promoting lifestyles.

**Results:**

The study found that factors such as medical majors (*t*_550_=2.47-7.55, *P*<.05) and greater concern with health (*t*_550_=2.15-9.01, *P*<.05) predicted college students’ 4-6 health-promoting lifestyle dimensions and the 3 dimensions of eHealth literacy. Moreover, critical eHealth literacy positively predicted all 6 health-promoting lifestyle dimensions (*t*_547_=2.66-7.28, *P*<.01), functional literacy positively predicted 2 dimensions (*t*_547_=2.32-2.98, *P*<.05), and interactive literacy predicted only the self-actualization dimension (*t*_547_=2.81, *P*<.01).

**Conclusions:**

This study found that participants who majored in medical fields had greater concern with their health and frequently sought health information, exhibited better eHealth literacy, and had a positive health-promoting lifestyle. Moreover, this study showed that college students with a higher critical eHealth literacy engaged better in health-promoting activities than those with functional and interactive literacy.

## Introduction

Global health challenges have gained increasing attention in recent years. Improving national health is an international goal. Developing countries strive to promote longevity and a good quality of life for their people and regard this goal as a national competition. The US Department of Health and Human Services found that unhealthy behaviors and lifestyles were 2 important factors that lead to the 10 major causes of death and greatly affect people’s health in their daily lives [1].

A health-promoting lifestyle is an important strategy to achieve public health. A health-promoting lifestyle, including self-actualization, health responsibility, exercise, nutrition, interpersonal support, and stress management, can be viewed as positive actions or perceptions directed toward maintaining or enhancing health and well-being [1]. A health-promoting lifestyle can help individuals attain positive health outcomes [2]. Therefore, understanding individuals’ health-promoting lifestyles can help us identify health problems and develop interventions to promote health.

Gillis reviewed the literature and found that health-promoting lifestyles are affected by individuals’ self-efficacy, social support, perceived benefits, and self-concepts as well as marital status, education, and knowledge about healthy lifestyles [3]. As health knowledge is based on health literacy, health literacy has been identified as a public health goal for the 21st century. The advent of the Internet has dramatically changed the landscape of health information; therefore, it is important to examine how eHealth literacy affects health-promoting lifestyles.

eHealth literacy is defined as the ability to seek, find, understand, and appraise health information from electronic sources and to apply the knowledge gained to address or solve health problems [4,5]. According to Nutbeam, health literacy can be divided into 3 levels: functional, interactive, and critical literacy. At the most basic level, functional literacy refers to basic reading and writing skills and the ability to apply basic literacy skills to health-related materials, such as reading the label on a pill bottle. Next is interactive literacy, which is predicated on functional health literacy and requires more advanced cognitive skills along with social skills that can be used to abstract information and derive meaning from different forms of communication. The highest level of critical literacy builds on functional and interactive literacy and involves the most advanced cognitive skills that can be applied to critically analyze information, discern the quality of health websites, and use quality information to make informed decisions about health. Together, interactive and critical health literacy involves complex skills that individuals use to extract, apply, evaluate, and analyze health-related information [6].

**Figure 1:**
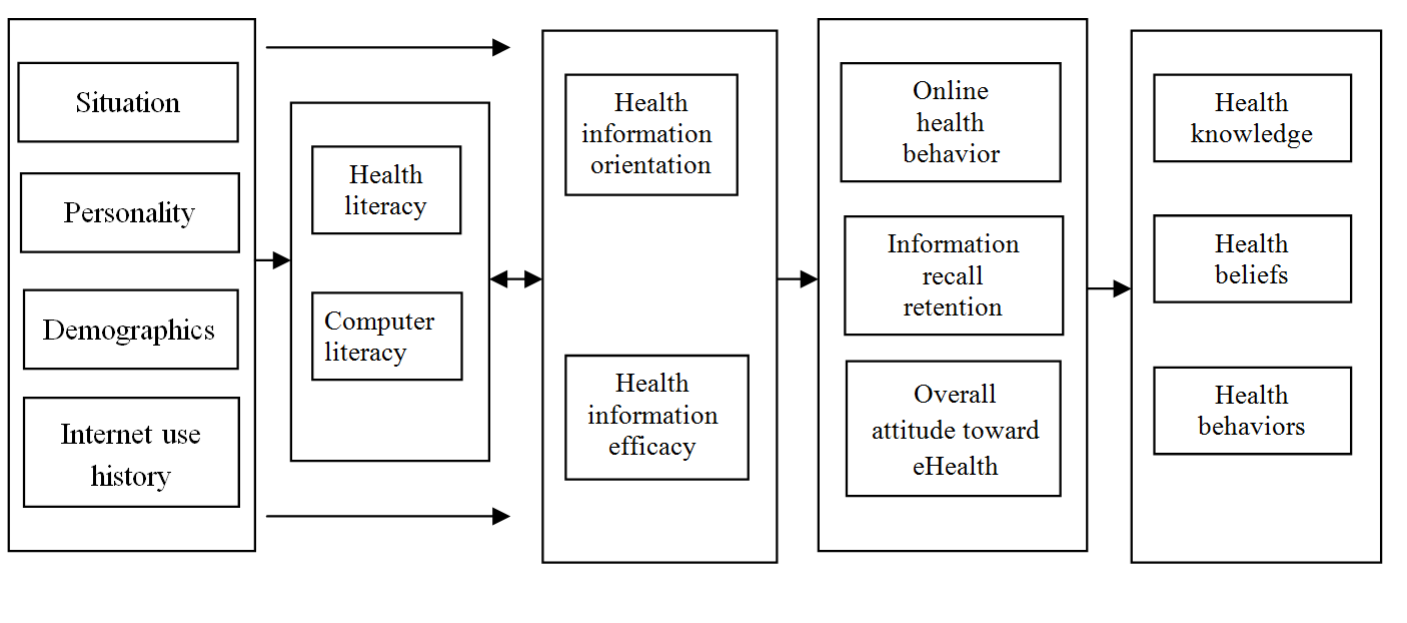
The integrative model of eHealth use.

eHealth literacy is becoming important in maintaining and promoting health. According to Pender’s health promotion model (HPM), each person has unique personal characteristics and experiences that affect subsequent health-promoting behaviors [7]. People who have better-developed health literacy will thus have skills and capabilities that enable them to engage in a range of health-enhancing actions [6]. The integrative model of eHealth use (IMeHU) suggests that people with high eHealth literacy are not only more inclined to use the Internet to find answers to health-related questions, but are also able to understand the information that they find, verify the veracity of the information, and use this information to promote health behaviors (Figure 1) [8].

Studies have found that the use of health information on the Internet affects personal exercise habits and eating or food consumption habits [9]. Similarly, studies have found that individuals with high eHealth literacy are more likely to adopt healthy eating, exercise, and sleep behaviors [5,10]. From the above study, it is clear that eHealth literacy affects physical health behaviors. A complete health-promoting lifestyle is composed of multiple dimensions, including psychological health behaviors (eg, self-actualization, health responsibility, interpersonal support, and stress management) [1,11,12]. However, few studies have examined the effect of individuals’ eHealth literacy on their psychological health behaviors. Thus, we adopt Pender’s HPM and IMeHU to explore the association between eHealth literacy and multiple types of health behaviors. A number of studies have found a positive relationship between health literacy and health-promoting behaviors [13-15]. For example, studies have found a significant association between health knowledge and health-promoting lifestyles among women of childbearing age [16]. In addition, eHealth literacy is actively promoted via health education. The eHealth intervention, which incorporates the functions and strategies of the eHealth interactive technology, encourages the adoption of physical and psychological behaviors among school health educators [17]. Accordingly, we propose the following hypothesis:

H1: College students who possess better eHealth literacy will engage in more positive health-promoting lifestyle behaviors.

Individuals’ health literacy and health behavior may be affected by their background such as education and situational characteristics related to health [18]. According to Pender’s HPM, an individual’s health concern is the factor that prompts the individual to adopt a health-promoting lifestyle. Previous studies have shown that individuals who prioritize their health or have numerous sources of health information tend to adopt more health-promoting lifestyles [19-21]. Some studies have also found that nurse training is significantly associated with health-promoting lifestyle behaviors [22]. Individuals who reported higher medical knowledge had better interpersonal relationships and lower levels of stress [23,24]. In addition, studies have found a positive link between organic food and an active lifestyle [25]. Accordingly, we propose the second hypothesis that is as follows:

H2: The individual factors of college students (eg, frequency of seeking information on health issues, degree of health concern, frequency of eating organic food, and students’ major) can predict their health-promoting lifestyles.

The IMeHU suggests that individual factors may affect an individual’s level of health and computer literacy as well as the individual’s perceived ability to use the Internet for health purposes [8]. Studies have found that college students who major in medical fields and who seek health information more frequently and have greater concern for their own health are more likely to have better eHealth literacy [5,26]. Organic foods are marketed as being healthier than conventional foods because organic food contains more antioxidants and lower levels of toxic metals and pesticide residues, and organic food consumers are more health conscious than other consumers are. Thus, it is inferred that individuals who consume organic food have better eHealth literacy. Accordingly, we propose the third hypothesis that is as follows:

H3: The individual factors of college students (eg, frequency of seeking information on health issues, degree of health concern, frequency of eating organic food, and students’ major) can predict eHealth literacy.

As a result, this study aimed to analyze whether the backgrounds of college students in Taiwan predict their eHealth literacy and health-promoting lifestyles and whether students’ eHealth literacy predicts a health-promoting lifestyle. These findings may have implications for health education for college students’ care and the popularization of national health policies for college students.

## Methods

### Participants

#### Sample for the Pretesting

It is important to test survey instruments before using them to collect data. Pretesting can help authors identify questions that do not make sense to participants, as well as problems with questionnaires that might lead to biased answers. Thus, pretesting was conducted to develop and test the adequacy of the research instrument designed by the authors. Exploratory factor analysis was used to assess the reliability of the survey instrument. For the pretest sample, a purposive sample of 250 college students was drawn from 3 schools in Taiwan. Each participant was mailed a questionnaire, and 207 usable (completed) questionnaires were returned, resulting in an effective response rate of 82.8%.

#### Sample for the Formal Study

This study was a cross-sectional study in Taiwan. We recruited 700 college students from 14 schools to participate in the survey in December 2015. The participants completed the questionnaire in their schools. After eliminating the respondents who had not completed the entire survey or who gave invalid responses, 556 valid surveys (79.4%) were retained. Among these 556 valid respondents, 207 (37.2%) studied in the northern region of Taiwan, 154 (27.7%) studied in the central region of Taiwan, 170 (30.6%) studied in the southern region of Taiwan, and 25 (4.5%) studied in the eastern region of Taiwan.

### The Survey Instrument

#### Individual Factors

We gathered the respondents’ individual factors, including the information about the frequency of seeking information on health issues, the frequency of eating organic food, the degree of health concern, and the students’ major. The frequency of seeking information on health issues and eating organic food was measured by asking how often the students sought general health-related information and ate organic food and was rated based on the responses on a scale from 1 (never) to 5 (always).

The students’ degree of health concern was measured by 1 item asking about their perception of health concerns (“Are you concerned about your health?”) and was rated on a scale from 1 (strongly unconcerned) to 5 (strongly concerned).

The major dimension was divided into the participants who were majoring in medical fields and the participants who were not. In a subsequent analysis, the 2 groups were transformed into dummy variables. We used the nonmedical group as the reference group.

#### eHealth Literacy Scale

eHealth Literacy was assessed by Chiang et al’s eHealth Literacy Scale (EHLS), which has been validated for Taiwan college students. The 3 subscales can be distinguished and show good internal consistency [10]. The individual item reliability of the 12-item EHLS ranged from 0.36 to 0.74. The standardized factor loadings ranged from 0.60 to 0.86 (*P*<.001). Composite reliability ranged from 0.75 to 0.84, and the average variance extracted for each dimension ranged from 0.50 to 0.52.

The EHLS is a reliable and valid measure of functional eHealth literacy (3 items), interactive eHealth literacy (4 items), and critical eHealth literacy (5 items). Answers were given on a 5-point Likert scale ranging from 5 (total agreement) to 1 (total disagreement). Mean scores for eHealth literacy scale were calculated by summing the item scores divided by the total number of items, resulting in a score ranging from 1 (lower eHealth literacy) to 5 (higher eHealth literacy).

#### Health-Promoting Lifestyle Scale

The Health-Promoting Lifestyle Scale (HPLS) was developed following a thorough review of the literature [1,11,12]. It contains 6 dimensions, which are as follows:

1. Self-actualization: attitudes toward and expectations of life (5 items, eg, willingness to try new things and challenges).

2. Health responsibility: paying attention to and taking responsibility for one’s own health (4 items, eg, discussions about health-related issues with health professionals).

3. Interpersonal support: a sense of intimacy and close relationships (4 items, eg, maintaining good relationships with others).

4. Exercise: regular exercise patterns (4 items, eg, exercise at least three times a week).

5. Nutrition: meal patterns and food choices (3 items, eg, intake of fiber-rich foods).

6. Stress management: ability to cope with stress (3 items, eg, a good balance between work and life). The items were answered using a 5-point Likert scale with scores ranging from 1 (never) to 5 (always).

An exploratory factor analysis (principal components extraction) revealed that the Kaiser-Meyer-Olkin test value was .89 (χ^2^_253_＝2528.70, *P*<.05), Bartlett sphericity test was significant (*P*<.05), factor loadings ranged from 0.59 to 0.81, and the explained variance was 69.8%. The high Cronbach alpha coefficients (0.86 for self-actualization, 0.87 for health responsibility, 0.84 for interpersonal support, 0.87 for exercise, 0.75 for nutrition, 0.71 for stress management, and 0.92 for the total scale) demonstrated high internal consistency.

### Data Analysis

First, a peer review was used to confirm the content validity of the HPLS. Second, exploratory factor analysis was used to assess the reliability of the HPLS. Third, 3 multiple regression analyses were used to examine the effects of individual factors on the 3 dimensions of eHealth literacy. Finally, 6 hierarchical multiple regression analyses were performed to examine the predictive variables on 6 dimensions of health-promoting behaviors. The researcher determined the order of the variables entered into a model based on logical or theoretical considerations. In step 1, individual factors were entered. In step 2, 3 dimensions of eHealth literacy were entered.

### Ethical Considerations

The study was reviewed and approved by the Institute of Education at the at the National Sun Yat-Sen University. The study adopted an anonymous questionnaire, in line with our government’s institutional review board rules of exempt review. The questionnaire instructions informed the participants of the research purpose and confidentiality and that they had the right to refuse to participate in the study at any time. The participants received the questionnaire and gifts at the same time. Even if a participant decided to drop out of the investigation, he or she still received the gifts. This approach was intended to be fair to each participant, to avoid the impact of gift inducements on the participants, and to serve as a compensation for the participants.

## Results

### Participant Demographics and Characteristics

Table 1 presents the demographics and characteristics of the study participants. Of the 556 participants, 80.9% were female. In terms of the participants’ majors, less than 20% of the participants majored in medical fields. Although a relative majority of the participants (43.5%) reported that their degree of health concern was average, an absolute majority of the participants (98.2%) reported that they had the experience of seeking health-related information. In terms of organic food, approximately 90% of the participants reported that they had experience eating organic foods.

**Table 1:** Sociodemographic and health information of the sample (N=556).

Variable and group		n (%)
**Gender**		
	Male	106 (19.1)
	Female	450 (80.9)
**Degree of health concern**		
	Strongly unconcerned	4 (0.7)
	Unconcerned	39 (7.0)
	Average	242 (43.5)
	Concerned	199 (35.8)
	Strongly concerned	72 (12.9)
**Major**		
	Major in medical field	106 (19.1)
	Major in nonmedical field	450 (80.9)
**Frequency of seeking information on health-related issues**		
	Never	10 (1.8)
	Seldom	125 (22.5 )
	Sometimes	270 (48.6)
	Often	116 (20.9)
	Always	35 (6.3)
**Frequency of eating organic food**		
	Never	60 (10.8)
	Seldom	199 (35.8)
	Sometimes	202 (36.3)
	Often	71 (12.8)
	Always	24 (4.3)

### Hierarchical Multiple Regression Analysis of the Variables Predicting a Health-Promoting Lifestyle

The results of hierarchical multiple regression analysis are displayed in Multimedia Appendix 1 (Model 1) and Multimedia Appendix 2 (Model 2). Multimedia Appendix 1 indicates that the individual factors positively predicted 6 dimensions of a health-promoting lifestyle, with a moderate level of predictive explanatory power for health responsibility (30%), exercise (23%), and nutrition (22%) and a low level of predictive explanatory power for self-actualization (17%), interpersonal support (16%), and stress management (15%). Notably, health concern positively predicted all 6 health-promoting lifestyle dimensions, and students’ major predicted the 4 health-promoting lifestyle dimensions. Both frequent health information seekers and organic food consumers emerged as predictors of health responsibility and exercise. Multimedia Appendix 2 shows that when controlling for the individual factors, critical eHealth literacy positively predicted all 6 health-promoting lifestyle dimensions. Functional eHealth literacy positively predicted the self-actualization and interpersonal support dimensions, whereas interactive eHealth literacy predicted only the self-actualization dimension. Among the 3 dimensions of eHealth literacy, critical eHealth literacy emerged as the best indicator.

### Multiple Regression Analysis of Individual Factors Predicting eHealth Literacy

Multimedia Appendix 3 indicates that all the individual factors except organic food consumption positively predicted the 3 dimensions of eHealth literacy, yielding low (functional adjusted *R*^2^*=*.14) and medium (Interactive: adjusted *R*^2^*=*.22, critical: adjusted *R*^2^*=*.20) predictive explanatory powers. In functional literacy, students’ major emerged as a strong predictor, frequently seeking information on health issues emerged as the strongest predictor of interactive literacy, and both greater health concerns and frequently seeking information on health issues emerged as the strongest predictors of critical literacy.

## Discussion

### Principal Findings

This study found that the participants who majored in medical fields had a greater concern for their health and frequently sought health information, exhibited better eHealth literacy, and had a positive health-promoting lifestyle. Moreover, participants who possessed better critical eHealth literacy engaged in more positive health-promoting lifestyle behaviors.

#### Lower Influence of Functional and Interactive Than Critical eHealth Literacy on Health-Promoting Lifestyles

Health literacy is a cognitive skill to empower individuals to take responsibility for their health and to adopt an appropriate lifestyle to keep themselves healthy resulting in personal benefit [6,27]. According to IMeHU, the promotion of individual eHealth literacy influences an individual’s health decision making and subsequently influences future actions that may help achieve better health [8]. The findings of the study showed that individuals with adequate health literacy have the knowledge and ability to make healthy choices and adopt healthy lifestyles to engage in a range of physical and mental health-enhancing actions [28]. Thus, Hypothesis 1 was partly supported. However, the study found that individuals with higher critical eHealth literacy engage in more health-promoting activities than those with functional and interactive literacy. In particular, critical literacy affects all dimensions of health-promoting behaviors. Critical literacy allows individuals to evaluate health issues and recognize risks and benefits as well as to advocate for themselves [29], thus enabling college students to engage in health-enhancing actions.

Notably, the finding of a lower influence of functional and interactive than critical eHealth literacy on health-promoting lifestyles is quite reasonable. Functional and interactive literacy are basic levels and the processing involved in functional and interactive eHealth literacy does not engage as deeply with issues as critical eHealth literacy do. According to the involvement theory [30], critical literacy is a more advanced cognitive skill than functional and interactive literacy. It is not sufficient for individuals to obtain health information; they must further evaluate and use the information to make decisions about their health. This may explain why functional and interactive eHealth literacy are less influential than critical eHealth literacy.

#### Individuals With Medical Majors and Greater Health Concern Might Have More Positive Health-Promoting Lifestyles

Consistent with Pender’s HPM and some other previous studies [19,21-24], this study found that participants with greater health concerns tended to adopt all 6 positive health-promoting lifestyles, with medical majors adopting 4 health behaviors (with the exception of exercise and nutrition). Individuals who frequently sought health information demonstrated better exercise and health responsibility. The findings largely supported the Hypothesis 2. The findings are also consistent with the social cognitive theory, suggesting that individuals’ medical knowledge can prompt individuals to adopt health-promoting behaviors. As medical school students have better cognitive understanding and perceptions of health information than nonmedical majors do, they are more willing to engage in appropriate health behaviors.

#### Individuals Who Are Medical Majors, Have Greater Health Concern, and Frequently Seek Health Information Might Have Better eHealth Literacy Development

Consistent with previous studies [5,10,26], our findings revealed that participants with medical majors and greater health concerns and those who frequently sought health information tended to have better functional, interactive, and critical eHealth literacy than other students did. Therefore, Hypothesis 3 was largely supported. The findings verified Bodie and Dutta’s IMeHU [8], indicating that eHealth literacy is influenced by a person’s educational background, intrinsic interest in health, and Internet use history. Medical school students have more medical knowledge [31] and therefore possess greater eHealth literacy than nonmedical school students. Individuals who have greater health concern and frequently use Web-based health resources are likely to pay more attention to their health and thus are likely to increase their eHealth literacy.

#### Organic Food Consumption Predicts a Health-Promoting Lifestyle, But Not eHealth Literacy

This study found that college students who frequently consumed organic food demonstrated better exercise and health responsibility than other students. Previous studies have shown that insufficient information and knowledge about organic labeling affects the distinction between organic food and conventional food [32]. Customers who consume organic food may need to understand the attributes and standards of organic food through greater efforts such as involvement in related information and discussion with relevant professionals. Consequently, it is reasonable that college students who frequently consume organic food demonstrate better health responsibility. In addition, some studies indicate a strong link between organic food choices and perceived healthfulness, well-being, and quality of life [33]. Regular consumers of organic food may have a high internal locus of control and naturally pay more attention to the positive benefits of food to maintain a healthy lifestyle [25,34]. Therefore, these consumers are ready to adopt healthy actions such as regular exercise and are accountable for their own personal health. It is also likely that people who are physically active pay more attention to their health and therefore buy organic food.

The study found that the frequency of organic food consumption did not predict the nutritional dimension. Previous studies lack strong evidence that organic foods are significantly more nutritious than conventional foods, and a number of studies have revealed that consumers’ choice of organic food depends on the perceived benefits of organic food [25,35,36]. Researchers have found that health is one of the most prominent motives for organic food consumption [25]. The perceived benefits of food safety, environmental protection, quality, and consumers’ perceptions of and attitudes toward labeling systems, message framing, and local origin are also identified as motivating factors for the consumption of organic food. The Taiwan food scandal involved a series of food safety incidents that came to light in 2014. This situation may have further strengthened Taiwan college students’ motivation to purchase organic food for food safety and reasons other than nutrition. Moreover, they could obtain appropriate nutrients via a balanced diet. Future studies might examine additional factors that characterize college students’ preferences and behavior toward organic food products.

The study found that the frequency of organic food consumption did not predict the nutrition dimension. According to previous studies, price is a factor that influences organic food consumption. Compared with conventional food, the overpricing of organic food can be considered an important barrier to the purchase desire of consumers [37,38]. Moreover, studies lack strong evidence that organic foods are significantly more nutritious than conventional foods [25]. Therefore, given the high price and uncertain benefits of organic food, students do not necessarily consume organic food to achieve the nutritional aspects of healthy living such as the intake of fiber-rich foods.

Notably, the frequency of organic food consumption did not predict any dimensions of eHealth literacy. This result may be caused by food safety awareness. Various food safety incidents worldwide have increased consumers’ concern about the safety of foods and are considered primary reasons for the increasing demand for organic food, which is perceived as healthier and safer [32,39]. Regardless of their level of health literacy, customers who buy organic products may be affected by their perceptions of the safety of the food rather than by the knowledge of organic food. Furthermore, eHealth literacy involves a complex interplay of basic literacy skills, the ability to successfully navigate the dominant language framework (English) and culture utilized for Web-mediated communication, and sufficient levels of technology adoption and proficiency [40]. When consumers shop for organic food in Taiwan, they need to look for the “CAS Organic” label. However, the label does not provide knowledge about the food, food sources, and nutritional facts. If consumers want to understand the nutritional facts of organic food, they need to seek, identify, understand, and use information about organic food. However, more than 90% of Web-based content is in English and is developed from the cultural perspectives of English speakers [41]. It is difficult for speakers of English as a second language to understand, extract, and evaluate eHealth information about organic food.

College students may have a positive attitude toward organic foods and high eHealth literacy but may not consume natural foods due to the higher price of these foods or a lack of convenience. Most Taiwan college students live on campuses, where the accommodations are not suitable for cooking, and they often eat restaurant food. In addition, there are few organic food courts. Even if students cook for themselves, they may not be able to afford organic food, which is more expensive than other food.

Therefore, the relationships among eHealth literacy, health-promoting behaviors, and organic food consumption require further studies to identify other mediating variables.

### Limitations

This study did not gather respondents’ variables such as age, school type, parental marital status, socioeconomic status, and health status. The analysis would have been stronger if the relationship between eHealth literacy and health-promoting lifestyles were also investigated while controlling for these individual factors. Moreover, this study found that functional and interactive eHealth literacy positively predicted 1-2 health-promoting lifestyle dimensions. There may be other mediating or confounding variables that should be taken into consideration. In addition, given that the factors that influence health-promoting lifestyles are complex and interdependent, future studies should explore which factors are critical and how these factors influence one another. For example, self-efficacy has been found to be a significant predictor of health-promoting lifestyles [2,42]. Future studies could further examine whether the measurement of self-efficacy and other critical factors add value to our understanding of the pathway from eHealth literacy to perceptions of health-promoting lifestyles. Notably, this study found that organic food consumption does not predict health promotion and eHealth literacy as much as students’ majors and health concerns do. Studies could consider other mediating or confounding variables such as food safety, organic food price, and attitude toward consumption. Further studies may utilize more integrative theories to study the factors involved in consumers’ choice of organic food and how they relate to eHealth literacy and health-promoting lifestyles.

### Conclusions

This study extends the previous research by identifying the associations among individual factors, eHealth literacy, and health-promoting lifestyles. The findings of our study corroborate the importance of individual factors in the occurrence of health-promoting behaviors and support the theoretical relationships among the concepts of the health-promotion model. In particular, greater health concern and medical majors affect eHealth literacy and health-promoting lifestyles. Given that greater health concern and medical majors are strong predictors of health-promoting behaviors and eHealth literacy, interventions to strengthen the importance and value of health and to enrich medical knowledge must be considered in programs for health improvement.

Previous studies have identified a positive change in health literacy and healthy lifestyle behaviors as the result of the education process [17,22]. Thus, by “learning health” to “live healthy,” school and government authorities can design appropriate programs to provide a much-needed repertoire of proven strategies to help students promote their eHealth literacy and maintain healthy lifestyles.

Moreover, this study showed that college students with higher critical eHealth literacy engage better in health-promoting activities than students with functional and interactive eHealth literacy. Thus, health education should aim not only to enhance basic reading and writing in functional literacy, but should also empower students with critical literacy to develop the skills, knowledge, and efficacy to act on that knowledge and maintain good health via participatory and critical approaches.

## Appendix

Multimedia Appendix 1 Hierarchical multiple regression analysis of 6 dimensions of a health-promoting lifestyle (Model 1).

Multimedia Appendix 2 Hierarchical multiple regression analysis of 6 dimensions of a health-promoting lifestyle (Model 2).

Multimedia Appendix 3 Multiple regression analysis of individual factors predicting eHealth literacy.

## Abbreviations

EHLS: eHealth Literacy Scale
HPLS: Health-Promoting Lifestyle Scale
HPM: health promotion model
IMeHU: integrative model of eHealth use
