# Supplementary material for: The Associations Among Individual Factors, eHealth Literacy, and Health-Promoting Lifestyles Among College Students
Source: J Med Internet Res. 2017 Jan 10;19(1):e15. doi: 10.2196/jmir.5964 (PMC5263862; doi:10.2196/jmir.5964)
Supplement: Multimedia Appendix 1 [file jmir_v19i1e15_app1.pdf]

Variable	Self-actualization				Health responsibility				Interpersonal support				Exercise				Nutrition				Stress management			
	B	Beta	t_{550}	*P*	B	Beta	t_{550}	*P*	B	Beta	t_{550}	*P*	B	Beta	t_{550}	*P*	B	Beta	t_{550}	*P*	B	Beta	t_{550}	*P*
Gender	.10	.01	.35	.73	.55	.07	1.96	.05	.20	.03	.88	.38	.80	.10	2.56	.01	.12	.02	.54	.59	.36	.07	1.73	.09
Seeking health issues	.17	.05	.91	.37	1.30	.34	7.30	<.001	.20	.07	1.33	.19	.80	.20	4.02	<.001	.17	.06	1.21	.23	.06	.02	.46	.65
Consuming organic food	−.15	−.05	−1.04	.30	.43	.13	3.20	.001	−.17	−.07	−1.52	.13	.41	.11	2.74	.006	.09	.04	.88	.38	.06	.03	.60	.55
Health concern	1.36	.36	7.65	<.001	.40	.10	2.29	.02	1.01	.33	7.06	<.001	1.17	.28	6.08	<.001	1.20	.41	9.01	<.001	.91	.34	7.13	<.001
Majors	1.21	.15	3.81	<.001	1.72	.20	5.60	<.001	.97	.15	3.82	<.001	−.47	−.05	−1.36	.17	.36	.06	1.50	.13	.57	.10	2.50	.01
	R=.42 $\Delta^a R^2$=.17				R=.55 ΔR^2=.30				R=.41 ΔR^2=.16				R=.48 $\Delta^a R^2$=.23				R=.47 ΔR^2=.22				R=.39 ΔR^2=.15			
	$F_{5,550}$=23.76				$F_{5,550}$=48.57				$F_{5,550}$=22.14				$F_{5,550}$=33.26				$F_{5,550}$=31.57				$F_{5,550}$=20.27			

[a]Δ: Adjusted.
