# Supplementary material for: The Associations Among Individual Factors, eHealth Literacy, and Health-Promoting Lifestyles Among College Students
Source: J Med Internet Res. 2017 Jan 10;19(1):e15. doi: 10.2196/jmir.5964 (PMC5263862; doi:10.2196/jmir.5964)
Supplement: Multimedia Appendix 2 [file jmir_v19i1e15_app2.pdf]

Variable	Self-actualization				Health responsibility				Interpersonal support				Exercise				Nutrition				Stress management			
	B	Beta	t_{547}	*P*	B	Beta	t_{547}	*P*	B	Beta	t_{547}	*P*	B	Beta	t_{547}	*P*	B	Beta	t_{547}	*P*	B	Beta	t_{547}	*P*
Gender	−.05	−.01	−.19	.85	.43	.06	1.58	.11	.09	.02	.44	.66	.72	.07	2.32	.02	.04	.01	.17	.87	.27	.05	1.37	.17
Majors	.54	.07	1.77	.08	1.64	.19	5.14	<.001	.43	.07	1.74	.08	−.47	−.05	−1.31	.19	.25	.04	1.03	.30	.39	.07	1.65	.10
Seeking health issues	−.32	−.09	−1.81	.07	1.07	.28	5.89	<.001	−.17	−.06	−1.22	.23	.67	.15	3.24	.001	−.01	−.00	−.06	.95	−.14	−.06	−1.61	.29
Health concern	.97	.25	5.80	<.001	.23	.06	1.30	.19	.67	.22	5.05	<.001	1.04	.25	5.31	<.001	1.06	.36	7.94	<.001	.75	.28	5.87	<.001
Consuming organic food	−.11	−.03	−.84	.40	.43	.13	3.24	.001	−.17	−.05	−1.22	.22	.41	.11	2.72	.007	.09	.04	.91	.36	.07	.03	.71	.48
Functional	.11	.09	2.32	.02	−.10	−.07	−1.97	.05	.12	.12	2.98	.003	−.07	−.05	−1 30	.19	−.05	−.05	−1.22	.23	−.02	-.02	−.41	.69
Interactive	.16	.14	2.81	.005	.11	.09	1.88	.06	.08	.09	1.83	.07	.03	.02	.42	.68	.07	.08	1.54	.13	.08	.09	1.70	.09
Critical	.30	.32	6.68	<.001	.16	.17	3.55	<.001	.26	.35	7.28	<.001	.14	.13	2.66	.008	.13	.18	3.56	<.001	.14	.22	4.15	<.001
	R=.57 $\Delta^{a}R^2$=.32				R=.59 ΔR^2=.34				R=.57 ΔR^2=.31				R=.50 ΔR^2=.24				R=.52 ΔR^2=.25				R=.46 ΔR^2=.20			
	$F_{8,547}$=33.02				$F_{8,547}$=36.71				$F_{8,547}$=32.09				$F_{8,547}$=22.82				$F_{8,547}$=24.66				$F_{8,547}$=18.67			

[a]Δ: Adjusted.
