# Supplementary material for: The Associations Among Individual Factors, eHealth Literacy, and Health-Promoting Lifestyles Among College Students
Source: J Med Internet Res. 2017 Jan 10;19(1):e15. doi: 10.2196/jmir.5964 (PMC5263862; doi:10.2196/jmir.5964)
Supplement: Multimedia Appendix 3 [file jmir_v19i1e15_app3.pdf]

Variable	Functional				Interactive				Critical			
	B	Beta	t_{550}	P	B	Beta	t_{550}	P	B	Beta	t_{550}	P
Gender	−.18	−.03	−.77	.44	.28	.05	1.20	.23	.43	.05	1.37	.17
Seeking health issues	.36	.13	2.48	.01	1.07	.35	7.18	<.001	.91	.23	4.64	<.001
Consuming organic food	−.20	−.08	−1.82	.07	−.04	−.02	−.38	.71	−.07	−.02	−.45	.67
Health concern	.34	.11	2.36	.02	.31	.10	2.15	.03	1.04	.25	5.48	<.001
Major	1.92	.30	7.55	<.001	1.23	.18	4.78	<.001	.84	.10	2.47	.01
	R=.39 $\Delta^{a}R^2$=.14				R=.48 ΔR^2=.22				R=.45 ΔR^2=.20			
	$F_{5,550}$=19.11				$F_{5,550}$=32.83				$F_{5,550}$=28.11			

[a]Δ: Adjusted.
